# Purification of Polysaccharide Produced by the Haploid Yeast Strain of *Tremella sanguinea* and Its Antioxidant and Prebiotic Activities

**DOI:** 10.3390/molecules28145391

**Published:** 2023-07-13

**Authors:** Yichi Liu, Suo Chen, Jialan Zhang, Mengxiang Gao, Li Li

**Affiliations:** 1 College of Life Science, Yangtze University, Jingzhou 434025, China; 2 College of Animal Science, Yangtze University, Jingzhou 434025, China; 3 Institute of Food Science and Technology, Yangtze University, Jingzhou 434025, China

**Keywords:** *Tremella sanguinea*, the haploid yeast stage, polysaccharide, antioxidant, prebiotics

## Abstract

*Tremella sanguinea* is a traditional Chinese medicinal and edible mushroom. Polysaccharides from *Tremella* mushrooms have received increasing amounts of research attention due to their diverse pharmacological activities. In this study, via the incubation of basidiospores collected from fresh artificially cultivated basidiocarps of *T. sanguinea*, a haploid yeast strain of *T. sanguinea* was obtained, and it was found to be a typical loose-slime-forming yeast capable of producing a large amount of exopolysaccharides (EPS). Using DEAE-52 cellulose column chromatography and Sephadex G-100 gel permeation chromatography, the major polysaccharide, named TSPS-1, was separated and purified from the EPS produced by the haploid yeast strain of *T. sanguinea*. TSPS-1 was a homogeneous polysaccharide with a molecular weight of 2.5 × 10^3^ kDa and consisted of rhamnose, glucose, xylose, mannose and glucuronic acid at a molar ratio of 1: 0.7: 62.2: 24.6: 11.5. The bioactivity of the TSPS-1 polysaccharide was evaluated. The results show that TSPS-1 exhibited noticeable antioxidant activity by scavenging hydroxyl radicals (EC_50_ = 1.92 mg/mL) and superoxide radicals (EC_50_ = 1.33 mg/mL), and prebiotic activity by promoting the growth of different probiotic strains in the genus *Lactobacillus* and *Bifidobacterium*. These results suggest that the cultivation of the haploid yeast strain can be a promising alternative for the efficient production of valuable *T. sanguinea* polysaccharides with antioxidant and prebiotic potential.

## 1. Introduction

**Figure 1:**
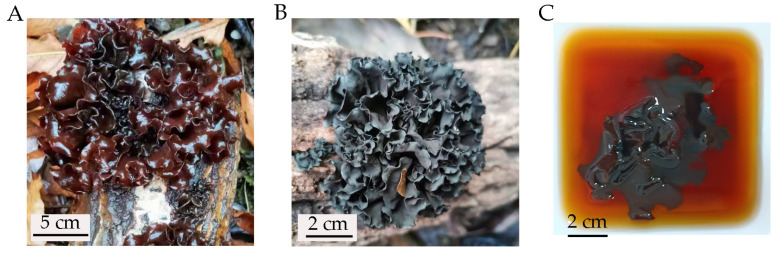
The basidiocarps of *Tremella sanguinea* on fallen logs. (**A**) Fresh basidiocarps; (**B**) dry basidiocarps; (**C**) pigments dissolved in water.

*Tremella sanguinea* is a type of edible jelly fungi that produces large, foliaceous, dark brown basidiocarps on fallen logs [1,2]. It is called “sanguine ear mushroom” as the basidiocarp is clumped in the form of ear flakes, and it oozes amber-red pigment when immersed in water (Figure 1). In China, *T. sanguinea*, together with *T. fuciformis* (silver ear mushroom) and *T. aurantialba* (golden ear mushroom), are the three most famous jelly mushrooms in the *Tremella* genus, which have been successfully cultivated and commercialized [1,3,4]. The pharmacological value of basidiocarps of *T. sanguinea* was recorded in the traditional Chinese medicine book “Compendium of Materia Medica” compiled by Li Shizhen in the Ming Dynasty (1368–1644), and has been used as a traditional Chinese medicine for the treatment of hepatitis, dysentery and gynecological diseases [2,5]. Recently, some chemical components with potential biological activities have been detected in the basidiocarps of *T. sanguinea*, including polysaccharides, steroids, alkaloids, lignans, fatty acids, and phenolic acid, among others [1,5], providing a strong chemical basis for its pharmacological value.

The edible mushrooms of the *Tremella* genus have been found to be extremely rich in polysaccharides, which are considered their main active ingredients [4,6]. Polysaccharides from *T. fuciformis* and *T. aurantialba* have been extensively investigated and have been demonstrated to have a variety of pharmacological activities, such as immunomodulatory, antitumor, antioxidant, anti-inflammatory, antiaging, antidiabetic, antiobesity and prebiotic activity [3,4,6,7,8]. Due to their diverse biological activities, polysaccharides from *T. fuciformis* and *T. aurantialba* have been widely used in medicine, and in the food and skin care industries [4,8]. Recently, polysaccharides from the basidiocarps of *T. sanguinea* have also been shown to have antioxidant, anti-inflammatory and immunomodulatory properties [1,9,10], revealing their potential future applications in healthcare products.

Currently, the artificial cultivation of the *T. sanguinea* mushroom in China still relies on the wild-like cultivation model. Due to the scarcity of *Tilia* sp. log substrate and the specific demands of the natural climate of mountainous areas between 900 and 1300 m in the subtropics, the annual production is extremely low (about 5000–8000 kg in Baokang Country, Hubei Province, which is the main production area of the mushroom *T. sanguinea* in China), limiting the further development and utilization of its functional ingredients such as *T. sanguinea* polysaccharides.

Fortunately, polysaccharides, the major group of bioactive ingredients in *Tremella*, have been extracted and isolated not only from the basidiocarps, but also from the fermentation culture of the isolated strains [3,6]. *Tremella* species are heterogeneous and there are two stages in their life cycle, the haploid yeast stage and the filamentous dikaryon stage; the haploid yeast stage is called tremellomycetous yeast [11,12,13]. It has been found that the basidiospore of both *T. fuciformis* and *T. aurantialba* could reproduce by budding and growing as haploid yeast cells [3,14,15,16]. As it is easier to achieve a high-density cultivation and shorter production period for yeast, the cultivation of the yeast form strain has been considered a more promising alternative for the large-scale production of polysaccharides from *T. fuciformis* [14,15,16] and *T. aurantialba* [3]. However, polysaccharides produced by the haploid yeast stage of *T. sanguinea* have not been reported. In this study, a haploid yeast strain of *T. sanguinea* was obtained via the incubation of basidiospores collected from fresh artificially cultivated basidiocarps of *T. sanguinea*. Then, a homogeneous exopolysaccharide was extracted and purified from the haploid yeast cells, and the chemical properties and biological activities of the polysaccharide were investigated.

## 2. Results and Discussion

### 2.1. Isolation of the Haploid Yeast Strain of T. sanguinea

**Figure 2:**
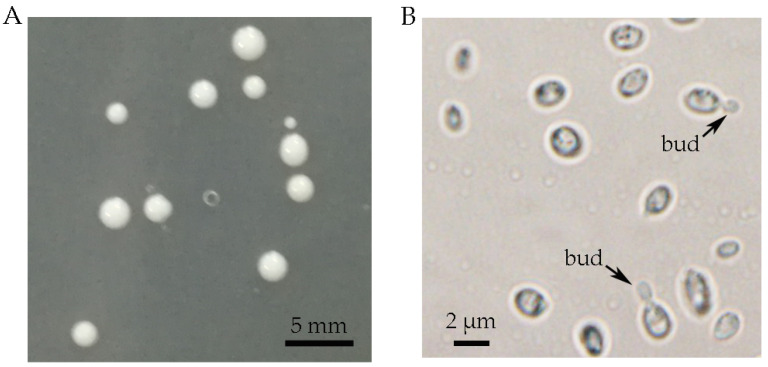
The colony morphology (**A**) and microscopic morphology (**B**) of the yeast-like isolates. The isolates were cultivated in PDA medium at 30 °C for 2 days.

**Figure 3:**
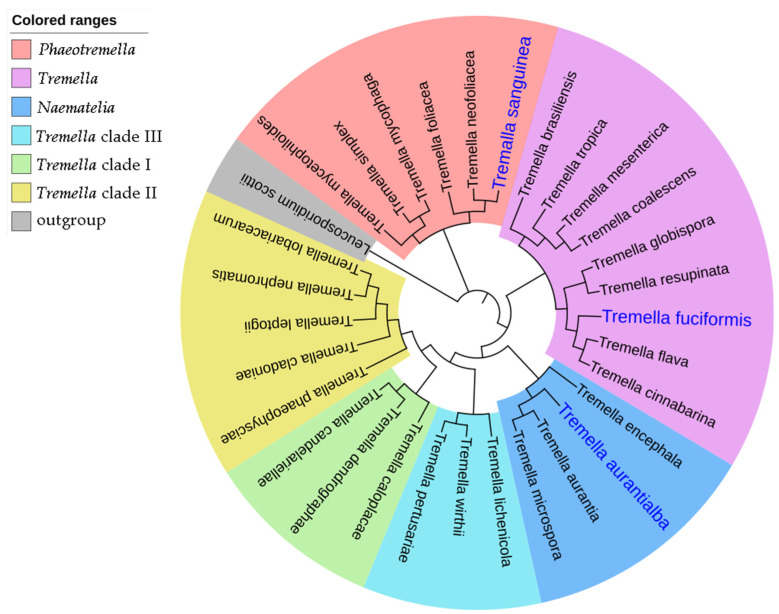
The phylogenetic tree based on ITS sequences. Branches in different colors represent different *Tremella* clades as described by Liu et al. [12]. *Leucosporidium scottii* was used as an outgroup. Accession numbers of nucleotide sequences are provided in Table 1.

After cultivating the basidiospores collected from fresh artificially cultivated basidiocarps of *T. sanguinea* on PDA plates at 30 °C for 2 days, yeast-like colonies with a similar appearance (round, convex, creamy-white and smooth) were observed (Figure 2A). In microscopic observation, the cells of the yeast-like isolates were ovoid, (1–2) μm × (2–3) μm, occurred singly or in pairs, and germinated via budding (Figure 2B). Four of the isolates were randomly selected for ITS sequencing (*GenBank* accession *number* MN894289, OR229791, OR229792 and OR229793), and the phylogenetic tree showed that they consistently fell within the *Phaeotremella* clade of genus *Tremella* (Figure 3), sharing a high sequence similarity with that of *T. neofoliacea* CCJ1204 (99%) and *T. foliacea* CBS 6969 (94%). The other two famous jelly mushrooms, *T. fuciformis* and *T. aurantialba,* fell within the *Tremella* clade and *Naematelia* clade (Figure 3), respectively, and shared a relatively lower sequence similarity with that of the yeast-like isolates (81% and 83%, respectively).

*T. sanguinea* was firstly reported in 1990, and identified as a new taxa belonging to the phylum of Basidiomycota, the class of Tremellomycetes, the order of Tremellales, and the family of Tremellaceae based on the morphological characteristics of the basidiocarps [17]. The Tremellomycetes contains a large number of dimorphic fungi with stable free-living unicellular yeast stage in their life cycles. These fungi have been conventionally classified as basidiomycetous yeasts [11]. The free-living yeast strains have been readily obtained from the basidiospores of many species of *Tremella*, including *T. fuciformis* [15] and *T. aurantialba* [3]. The morphological and phylogenetic features of the yeast-like isolates from basidiospores of *T. sanguinea* (Figure 2 and Figure 3) indicated that they should be the free-living unicellular yeast strains of *T. sanguinea*. Given that there were no apparent differences among these yeast-like isolates in the features observable through colony morphology, microscopic morphology and ITS sequences, one of the isolates, designated *T. sanguinea* strain XE01, was subjected to further investigation for its EPS in subsequent studies.

### 2.2. Extraction and Purification of EPS from the Haploid Yeast Strain of T. sanguinea

**Figure 4:**
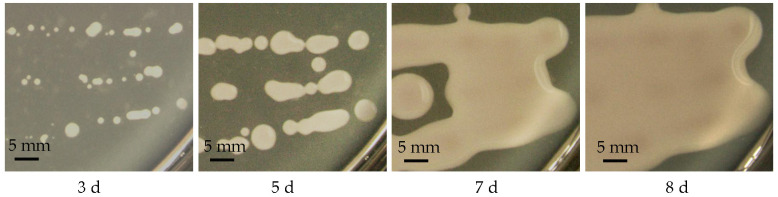
Development of large diffuse colonies with slimy appearance. The haploid yeast strain of *T. sanguinea* was cultivated on PDA plates at 30 °C for 8 days.

With the increase in incubation time, large diffuse colonies with a slimy appearance developed and then spread across the whole surface of PDA plates (Figure 4). After 8 days of cultivation, the mucoid culture of the haploid yeast strain of *T. sanguinea* was subjected to water extraction, which was followed by deproteinization, ethanol precipitation and lyophilization, and crude EPS were obtained with a yield of approximately 16.1 ± 1.1 g per 100 g of dry cells.

**Figure 5:**
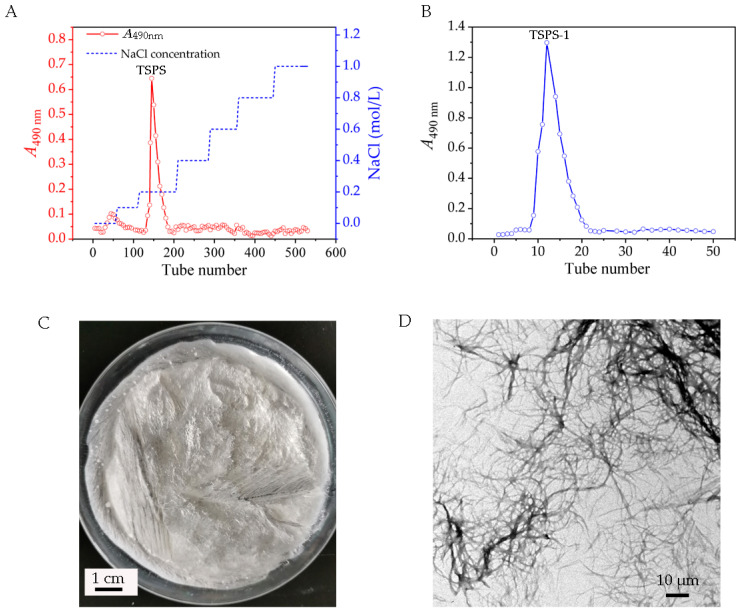
Purification of EPS from the haploid yeast strain of *T. sanguinea*. (**A**) Elution curve of DEAE-52 cellulose column chromatography; (**B**) elution curve of Sephadex G-100 gel permeation chromatography; (**C**) lyophilized polysaccharide TSPS-1; (**D**) TEM image of polysaccharide TSPS-1.

**Figure 6:**
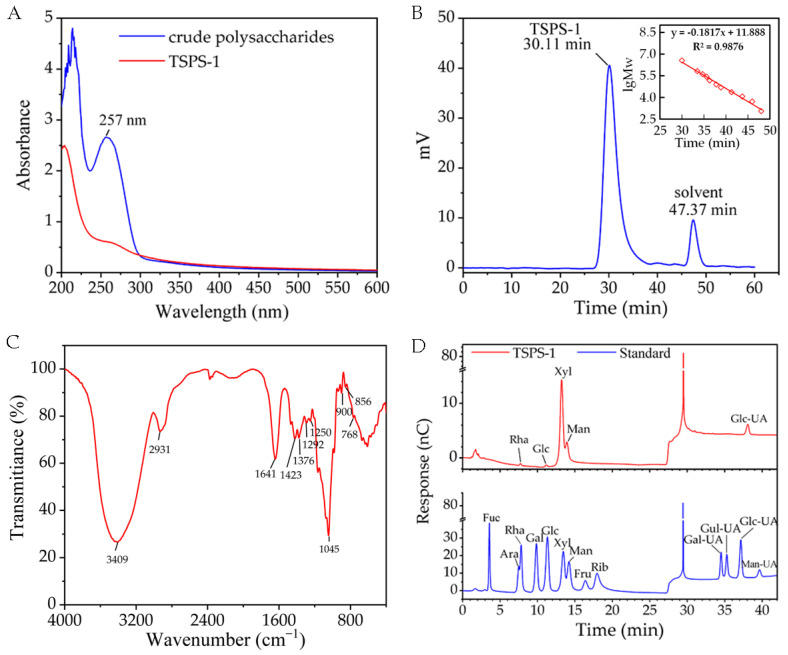
UV-Vis spectrum (**A**), HPGPC profile (**B**), FT-IR spectrum (**C**) and monosaccharide composition (**D**) of the polysaccharide TSPS-1.

DEAE-52 cellulose column chromatography of the crude EPS showed one major carbohydrate-positive peak (fractions #140–175), which was eluted at 0.2 mol/L NaCl (Figure 5A). This main fraction (named TSPS) was further purified using Sephadex G-100 gel permeation chromatography based on the molecular size, and a major fraction named TSPS-1 was obtained (Figure 5B). TSPS-1 was white in color and exhibited a molecule conformation in highly extended chain (Figure 5C,D). The TSPS-1 fraction was free of nucleic acid and protein as no distinct absorption peaks around 260 and 280 nm were shown in the UV–Vis spectrum (Figure 6A). The HPGPC profile showed a single and symmetrical sharp peak at 30.11 min (Figure 6B), indicating that TSPS-1 was homogeneous. The average molecular weight was about 2.5 × 10^3^ kDa according to the calibration curve with standard dextrans.

It is known that many microorganisms are capable of producing EPS material of a gelatinous or gummy consistency, and the EPS material may remain firmly adherent as a discrete layer surrounding the cell, that is, as a capsule or slime layer, or the material may part freely from the cell as loose or free slime [18,19,20]. Loose slime will normally remain as a confluent matrix around organisms in colonies on solid media, but usually disperses from the cell in a liquid medium [18]. Here, the haploid yeast strain of *T. sanguinea* was found to be a typical loose-slime-forming yeast (Figure 4) and to have the ability of producing a large amount of EPS, suggesting that the cultivation of the yeast stage strain would be an alternative for the large-scale production of *T. sanguinea* polysaccharides.

### 2.3. Structural Analysis of the Polysaccharide TSPS-1

The FT-IR spectra of TSPS-1 showed characteristic polysaccharide bands (Figure 6C). The strong and wide absorption of approximately 3409 cm^−1^ and weak absorption band of approximately 2931 cm^−1^ were attributed to O-H and C-H stretching vibrations, respectively, which were characteristic of sugars and derivatives [21]. The absorptions at 1641 and 1423 cm^−1^ were ascribed to asymmetric and symmetric C=O stretching vibrations of the carboxyl group [22], respectively, indicating that uronic acids existed in TSPS-1. The strong absorption of approximately 1045 cm^−1^ was attributed to the characteristic stretching vibration of C-O-C [7,23], which suggested that sugar rings of TSPS-1 were pyranose rings. In addition, the absorption peak at 768 cm^−1^ also confirmed the pyran structure because the weak absorption was due to the symmetrical stretching vibration of α-pyran [21]. The weak absorption bands in the region of 900 and 856 cm^−1^ implied that α- and β-glycosidic linkages existed simultaneously [21]. These results indicated that TSPS-1 was a kind of acid polysaccharide with a sugar ring of pyranose, linked by α- and β- configuration glycosidic bonds.

According to monosaccharide composition analysis (Figure 6D), TSPS-1 was a heteropolysaccharide and composed of rhamnose, glucose, xylose, mannose and glucuronic acid, with a molar ratio of 1:0.7:62.2:24.6:11.5, which was different from that of the polysaccharide purified from the basidiocarps of *T. sanguinea* [1]. The syntheses of microbial EPS are regarded as secondary metabolites, and their structure and physical properties have been found to be related to the physiological states of the producers [19,20]. The polysaccharides produced by *T. fuciformis* have also been found to have a molecular weight ranging from 5.82 × 10^5^ Da to 3.74 × 10^6^ Da [6]. In addition, different experimental conditions obtained different *T. fuciformis* polysaccharide fractions [6].

### 2.4. Antioxidant Activity of the Polysaccharide TSPS-1

**Figure 7:**
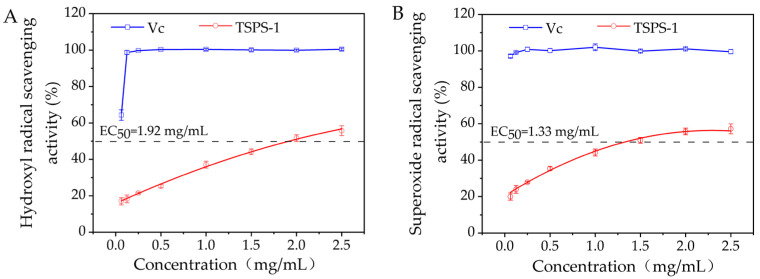
The hydroxyl radical (**A**) and superoxide radical (**B**) scavenging effect of the polysaccharide TSPS-1 at different concentration. Ascorbic acid (Vc) was used as a positive control.

The antioxidant activity of the polysaccharides was found to be related to the monosaccharide component, molecular size, structure and conformation [24]. The monosaccharides in the polysaccharides are reductive agents as they can supply hydrogen, which can combine with radicals to terminate the radical reaction. On the basis of hydroxyl radical assay and superoxide radical assay, the antioxidant activities of the polysaccharide TSPS-1 were investigated. As shown in Figure 7, the scavenging effect of TSPS-1 on the hydroxyl radical and superoxide radical increased dose-dependently and was 56% and 57% at a concentration of 2.5 mg/mL, respectively. The EC_50_ values of TSPS-1 for hydroxyl radical and superoxide radical were 1.92 mg/mL and 1.33 mg/mL, respectively. The above results suggest that the polysaccharide TSPS-1 was effective in antioxidation.

### 2.5. Effect of the Polysaccharide TSPS-1 on the Proliferation of Probiotics

**Figure 8:**
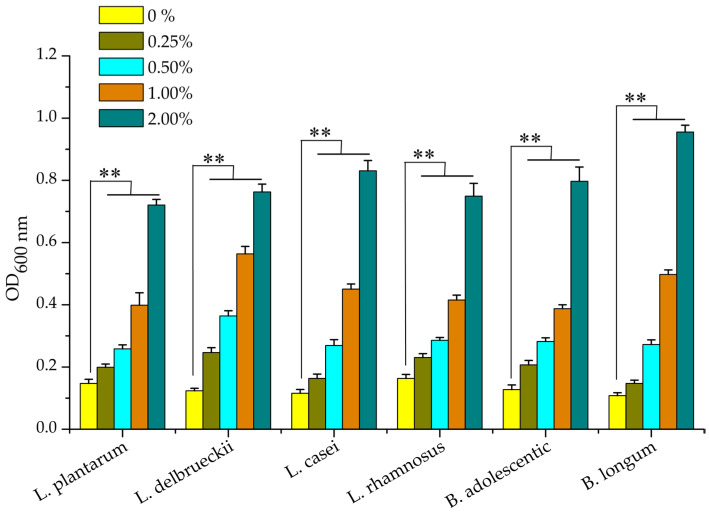
Effect of the polysaccharide TSPS-1 on the proliferation of probiotics. Rogosa broth media without glucose supplemented with 0.0–2.0% polysaccharide TSPS-1 was inoculated with strains of *L. plantarum*, *L. delbrueckii*, *L. rhamnosus*, *L. casei*, *B. longum* or *B. adolescentic*, and incubated for 48 h at 37 °C under anaerobic condition. ** represents a significant difference between samples (*p* < 0.05).

A number of studies indicate that non-starch polysaccharides are usually resistant to digestion in the human gastrointestinal tract owing to a lack of carbohydrate active enzymes in the human body; however, they are easily fermented by colonic microbiota in the intestine, resulting in the maintenance of the balance of micro-ecology and the diversity of intestinal microbiota [7,25]. Strains of *L. plantarum*, *L. delbrueckii*, *L. rhamnosus*, *L. casei*, *B. longum* and *B. adolescentic* were cultivated in Rogosa medium supplemented with 0.25–2.00% (W/V) of the polysaccharide TSPS-1 as the sole carbon source. As shown in Figure 8, the growth of all six probiotic strains was significantly promoted by polysaccharide TSPS-1 at a concentration range from 0.25% to 2.00% (*p* < 0.05). These results indicate that polysaccharide TSPS-1 possessed prebiotic potential and could promote the growth of different probiotic strains in the genus *Lactobacillus* and *Bifidobacterium*.

## 3. Materials and Methods

### 3.1. Isolation of the Haploid Yeast Strain of T. sanguinea

Artificially cultivated fresh basidiocarps of *T. sanguinea* were gathered from their main production area in China, which was located in Baokang Country, Hubei Province. The basidiospores of the fresh basidiocarps were collected using the spore-fall method and then spread on potato dextrose agar (PDA) supplemented with 200 mg/L chloramphenicol. After being cultivated at 30 °C for 2 days, the yeast-like colonies were purified via repeated streaking (at least three times) on the same medium. An OLYMPUS BX63 optical microscope (Olympus, Tokyo, Japan) was employed for the microscopic observation of isolates.

### 3.2. Phylogenetic Analysis

After culturing the yeast isolates in potato dextrose broth (PDB) for 2 days at 200 rpm, 30 °C, the genomic DNA was extracted using the Ezup column yeast genomic DNA purification kit (Sangon Biotech, Shanghai, China). The ITS region was amplified with fungus-specific universal primers ITS1 (5′-TCCGTAGGTGAACCTGCGG-3′) and ITS4 (5′-TCCTCCGCTTATTGATATGC-3′). The reaction was performed in a final volume of 50 μL including 100 ng genomic DNA, 25 µL 2 × High Fidelity PCR Master Mix (Sangon Biotech, Shanghai, China), and 2 µL forward and reverse primers (10 µM each). The reaction conditions were as follows: initial denaturation step at 95 °C for 3 min, 30 cycles of denaturation at 95 °C for 15 s, annealing at 56 °C for 15 s, extension at 72 °C for 1 min, and a final extension step at 72 °C for 10 min. The PCR products were purified using the EZ-10 Spin Column DNA Gel Extraction Kit (Sangon Biotech, Shanghai, China), sequenced by Sangon Biotech, Shanghai, China. The sequences acquired for the selected isolates were searched using BLAST against the GenBank nucleotide database to determine the similarity between the isolate sequences and the corresponding sequences of defined species. Then, the phylogeny of the acquired sequences was analyzed using MEGA X. A phylogenetic tree was reconstructed using the neighbor-joining (NJ) method with distances computed using the maximum composite likelihood evolutionary model and bootstrapped with 1000 replicates. The sequences used for phylogenetic analysis are listed in Table 1. *Leucosporidium scottii* was used as an outgroup.

### 3.3. Preparation of Crude Polysaccharides

The cell suspension (10^4^ cells/mL) of the isolated haploid yeast strain of *T. sanguinea* was spread on PDA plates and cultivated at 30 °C for 8 days. Then, the yeast cells in each plate were suspended in 20 mL of deionized water and collected for extraction by shaking (100 rpm) at 30 °C for 30 min. After centrifugation at 10,000 r/min for 15 min, the supernatant was concentrated to 1/5 of the original volume under vacuum at 55 °C. The concentrated extracts were further purified via deproteinization using the Savage reagent (chloroform/isoamyl alcohol, 4:1, *v*/*v*). Subsequently, the polysaccharides were precipitated with four volumes of 100% ethanol and kept overnight at 4 °C. The resulting precipitates of the crude polysaccharides were collected via centrifugation (10,000 r/min for 15 min) and lyophilized. The polysaccharide yields were calculated as described below: yield (%, w/w) = weight of extracted polysaccharides/weight of dried yeast cells × 100.

### 3.4. Purification of the Polysaccharide TSPS-1

The crude polysaccharides (0.1 g) was dissolved in 10 mL of deionized water, and then the samples were loaded into a DEAE-52 cellulose column (2.6 cm × 45 cm) equilibrated with a linear NaCl gradient (0–1.0 mol/L) at a flow rate of 60 mL/h. The polysaccharide fractions were collected and further purified via gel permeation chromatography on a Sephadex G-100 column (2.6 cm × 45 cm) eluted with deionized water at a flow rate of 30 mL/h. The main polysaccharide fraction was dialyzed against deionized water and subsequently lyophilized to obtain a homogeneous polysaccharide named as TSPS-1.

### 3.5. UV-Vis and FT-IR Analysis

The UV-Vis absorbance spectrum of the polysaccharide TSPS-1 solution (5 mg/mL) was recorded using a TU-1900 UV-Vis spectrophotometer (Presee, Beijing, China) in a range of 200 to 600 nm at 1 nm intervals.

The Fourier transform infrared (FT-IR) spectrum of the polysaccharide TSPS-1 was recorded using a IR-960 FT-IR spectrophotometer (Tianjin Ruian Technology Co., Ltd., Tianjin, China), with a 4000 to 400 cm^−1^ scanning range and a 4 cm^−1^ resolution. About 2 mg TSPS-1 was incorporated and pelletized with KBr.

### 3.6. Microscopic Analysis

The microstructure of the polysaccharide TSPS-1 was observed following the method reported by Wang et al. [1]. In brief, the aqueous solution of polysaccharide TSPS-1 was prepared at a concentration of 1 mg/mL, followed by the addition of an equal volume of SDS solution (1 mg/mL). The mixture was heated at 80 °C for 2 h, diluted with deionized water to a final concentration of 5 μg/mL, and then heated at 80 °C for 2 h. A droplet of this polysaccharide solution was deposited on the carbon film specimen (300 mesh). After drying at an ambient temperature, a transmission electron microscopy (TEM) (HT7800, Hitachi High-Technologies Corporation, Tokyo, Japan) with an accelerating voltage of 80 kV was applied to visualize the molecular morphology of the polysaccharide TSPS-1.

### 3.7. Determination of Molecular Weight

The relative molecular weight of the polysaccharide TSPS-1 was determined via high performance gel permeation chromatography (HPGPC) using a HPLC system (Waters 1515) equipped with a refractive index detector (RID, Waters 2410) and tandem chromatographic columns of Shodex OHpak SB-803 HQ, 804 HQ and 805 HQ columns (8.0 mm × 300 mm, Showa Denko K. K. Tokyo, Japan). The columns were eluted with a 0.05 mol/L NaCl solution at a flow rate of 0.6 mL/min. Dextrans (molecular weights of 1,152, 5,000, 11,600, 23,800, 48,600, 80,900, 148,000, 273,000, 409,800, 667,800, 3,693,000 Da) were used as the standard for molecular weight determination.

### 3.8. Analysis of Monosaccharide Composition

The monosaccharide composition of the polysaccharide TSPS-1 was determined using a Thermo ICS-5000 ion-exchange chromatography system equipped with an electrochemical detector and Dionex™ CarboPac™ PA20 column (3.0 mm × 150 mm, 10 μm). The polysaccharide TSPS-1 was dissolved in 2 mol/L of trifluoroacetic acid (TFA) and hydrolyzed at 121 °C for 2 h in sealed glass tubes. The hydrolysates were then dried under nitrogen flow to completely remove the remaining TFA and dissolved in deionized water for analysis. The standard monosaccharides used for analysis were fucose (Fuc), rhamnose (Rha), arabinose (Ara), galactose (Gal), glucose (Glc), xylose (Xyl), mannose (Man), fructose (Fru), ribose (Rib), galacturonic acid (Gal-UA), glucuronic acid (Glc-UA), mannuronic acid (Man-UA) and guluronic acid (Gul-UA) (Sigma-Aldrich, St. Louis, MO, USA).

### 3.9. Determination of Antioxidant Activities in Vitro

The hydroxyl radical-scavenging assay was measured using the method previously described by Zhang et al. [21]. Briefly, 1.0 mL of 9 mmol/L ferrous sulfate, 1.0 mL of 9 mmol/L salicylic acid-ethanol solution, and 1.0 mL polysaccharide TSPS-1 solution were mixed. To start the reaction, 1.0 mL of 8.8 mmol/L hydrogen peroxide was added, as the hydroxyl radicals were generated by reacting with the ferrous sulfate. The system was incubated in a water bath at 37 °C for 1 h. The absorbance of the reaction systems with various concentrations of polysaccharide TSPS-1 was measured at 510 nm with ascorbic acid (vitamin C, Vc) as the positive control (A_1_). The absorbance when salicylic acid-ethanol solution was replaced with 1.0 mL of ethanol was expressed as A_2_. The absorbance of the blank control in which the polysaccharide solution was replaced by 1.0 mL of deionized water was expressed as A_0_. The hydroxyl radical scavenging rate was calculated using the following equation: scavenging rate (%) = [1 − (A_1_ − A_2_)/A_0_] × 100.

Superoxide radical-scavenging assay was measured using the method previously described by Feng et al. [26]. Briefly, 3.0 mL of 50 mmol/L Tris-HCl buffer (pH 8.2) was incubated at 25 °C for 20 min, and then 2.0 mL of the polysaccharide TSPS-1 solution and 1.0 mL of pyrogallic acid (50 mmol/L) were added to the buffer. The mixture was rapidly mixed and incubated at 25 °C for 5 min. Subsequently, 0.2 mL HCl (1 mol/L) was added to the mixture to terminate the reaction. The absorbance of the reaction systems with various concentrations of polysaccharide TSPS-1 was measured at 325 nm with Vc as the positive control (A_1_). The absorbance when pyrogallic acid solution was replaced with 1.0 mL of deionized water was expressed as A_2_. The absorbance of the blank control in which the polysaccharide solution was replaced by 2.0 mL of deionized water was expressed as A_0_. The superoxide radical scavenging rate was calculated using the following equation: scavenging rate (%) = [1 – (A_1_ – A_2_)/A_0_] × 100.

### 3.10. Probiotic Bacteria Growth Stimulation

The potential of the polysaccharide TSPS-1 for the stimulation of the growth of probiotic strains was determined using the method previously described [27] with a minor modification. *Lactobacillus plantarum* subsp. plantarum (bio-61676, Biobw, Beijing, China), *L. delbrueckii* subsp. bulgaricus (bio-58490, Biobw, Beijing, China), *Bifidobacterium longum* ATCC 15707, *B. adolescentic* ATCC 15076, *L. rhamnosus* ATCC 7469 and *L. casei* CGMCC 1.29 were used as targets for the test. The determination of the growth of the six probiotic strains in the presence of the polysaccharide TSPS-1 was performed in U-shaped 96-well plates. Cultures of probiotic strains grown for 24 h at 37 °C under anaerobic condition in Rogosa broth media were adjusted in saline solution (0.85% NaCl) to an OD_600_ of 0.1 (approximately 1.5 × 10^8^ CFU/mL) for the seed culture. Rogosa broth media (without glucose, 200 µL) supplemented with 0.0%–2.0% polysaccharide TSPS-1 were inoculated with 20 µL of the seed culture, and incubated for 48 h at 37 °C under anaerobic condition. The absorbance was measured in an EnSpire Multimode Plate Reader (PerkinElmer, Norwalk, CT, USA) at a wavelength of 600 nm.

### 3.11. Statistical Analysis

All the experiments were repeated at least three times. The results were expressed as the mean over the three replicates. The data were analyzed using Origin 8.5 (OriginLab, Northampton, MA, USA). The significance of the difference (*p* < 0.05) was analyzed via one-way analysis of variance (ANOVA) and Duncan’s multiple range tests.

## 4. Conclusions

Using the spore-fall method, the basidiospores were collected from fresh artificially cultivated basidiocarps of *T. sanguinea* gathered from its main production area in China (Baokang Country, Hubei Province). After incubating the basidiospores in PDA medium, a *T. sanguinea* strain in the haploid yeast stage was obtained. The colonies of the haploid yeast strain were round, convex, creamy-white and smooth; their cells were ovoid, (1–2) μm × (2–3) μm and occurred singly or in pairs, germinated via budding. Phylogenetic analysis based on ITS sequences showed that the haploid yeast strain of *T. sanguinea* fell within in the *Phaeotremella* clade of the genus *Tremella*, and showed a closer phylogenetic relationship to *T. neofoliacea* and *T. eugeniae*.

After incubating the haploid yeast strain of *T. sanguinea* in PDA medium at 30 °C for 8 days, large diffuse colonies with a slimy appearance developed. Using procedures of water extraction, deproteinization, ethanol precipitation and lyophilization, EPS were obtained from the mucoid culture of the haploid yeast strain, with a yield of approximately 16.1 ± 1.1 g per 100 g dry cells. Using DEAE-52 cellulose column chromatography and Sephadex G-100 gel permeation chromatography, the major polysaccharide, named TSPS-1, was separated and purified from the EPS. TSPS-1 was a homogeneous acid polysaccharide with a molecular weight of 2.5 × 10^3^ kDa and consisted of rhamnose, glucose, xylose, mannose and glucuronic acid at a molar ratio of 1:0.7:62.2:24.6:11.5.

The bioactivity of the polysaccharide TSPS-1 was evaluated. The results show that TSPS-1 exhibited noticeable antioxidant activity by scavenging the hydroxyl radical (EC_50_ = 1.92 mg/mL) and the superoxide radical (EC_50_ = 1.33 mg/mL), and prebiotic activity by promoting the growth of different probiotic strains in the genus *Lactobacillus* and *Bifidobacterium* at a concentration range of 0.25%–2.00% (W/V), including *L. plantarum*, *L. delbrueckii*, *L. rhamnosus*, *L. casei*, *B. longum* and *B. adolescentic*.

The present research provided clues on the utility of haploid yeast strains of *T. sanguinea* for an efficient production of valuable *T. sanguinea* polysaccharides with antioxidant and prebiotic potential.

## Figures and Tables

**Table 1:** Sequences used for phylogenetic analysis.

Species	Strains	GenBank Numbers
Tremella mycetophiloides * (*Phaeotremella mycetophiloides*)	DSM 5728	MF580587.1
T. mycophaga * (*P. mycophaga*)	RJB6539-4	AF042431.1
T. simplex * *(P. simplex* *)*	FO31782	AF042428.1
T. foliacea * (*P. simplex*)	CBS 6969	AF444431.1
T. neofoliacea * (*P. simplex*)	CCJ1204	AF042415.1
*T. brasiliensis*	CBS 6966	AF444429.1
*T. mesenterica*	CBS 6973	AF444433.1
*T. tropica*	CCJ1355	AF042433.1
*T. coalescens*	CBS6967	KF036601.1
*T. resupinata*	CCJ1458	AF042421.1
*T. fuciformis*	CBS 6970	NR_155936.1
*T. cinnabarina*	CBS 8234	NR_155933.1
*T. globispora*	CBS 6972	NR_155889.1
*T. flava*	CBS 8471	NR_155935.1
*T. aurantia* * (*Naematelia aurantia*)	CBS 6965	NR_155873.1
*T. encephala* * (*N. encephala*)	CCJ925	AF042404.1
*T. microspora* * (*N. microspora*)	BPI702328	AF042435.1
*T. aurantialba* * (*N. aurantialba*)	9901	DQ400104.1
*T. lichenicola*	AM18	JN053504.1
*T. pertusariae*	AM2	JN053494.1
*T. wirthii*	AM90	JN053492.1
*T. caloplacae*	AM31	JN053468.1
*T. candelariellae*	AM34	JN053470.1
*T. dendrographae*	AM39	JN053471.1
*T. leptogii*	AM81	JN053476.1
*T. lobariacearum*	AM118	JN053474.1
*T. nephromatis*	AM133	JN053475.1
*T. phaeophysciae*	AM121	JN053480.1
*T. cladoniae*	AM84	JN053478.1
*Leucosporidium scottii*	CBS 5930	NR_073267.1

* Based on molecular phylogenetic analysis, classification for these *Tremella* species has been suggested to be updated, and the basionyms are presented in brackets.

## Data Availability

The datasets used and/or analyzed during the current study are available from the corresponding author on reasonable request.
